# Spacer Domain in Hepatitis B Virus Polymerase: Plugging a Hole or Performing a Role?

**DOI:** 10.1128/jvi.00051-22

**Published:** 2022-04-12

**Authors:** Caitlin Pley, José Lourenço, Anna L. McNaughton, Philippa C. Matthews

**Affiliations:** a School of Clinical Medicine, University of Cambridge, Cambridge, United Kingdom; b Guy’s and St Thomas’ NHS Foundation Trust, London, United Kingdom; c Department of Zoology, University of Oxford, Oxford, United Kingdom; d Biosystems and Integrative Sciences Institute, University of Lisbon, Lisbon, Portugal; e Population Health Science, Bristol Medical School, University of Bristol, Bristol, United Kingdom; f Nuffield Department of Medicine, University of Oxford Medawar Building, Oxford, United Kingdom; g The Francis Crick Institute, London, United Kingdom; h Division of Infection and Immunity, University College London, London, United Kingdom; Cornell University

**Keywords:** hepatitis B virus, HBV, spacer, evolution, diversity, polymorphism, polymerase, genotype, phylogeny

## Abstract

Hepatitis B virus (HBV) polymerase is divided into terminal protein, spacer, reverse transcriptase, and RNase domains. Spacer has previously been considered dispensable, merely acting as a tether between other domains or providing plasticity to accommodate deletions and mutations. We explore evidence for the role of spacer sequence, structure, and function in HBV evolution and lineage, consider its associations with escape from drugs, vaccines, and immune responses, and review its potential impacts on disease outcomes.

## INTRODUCTION

**FIG 1:**
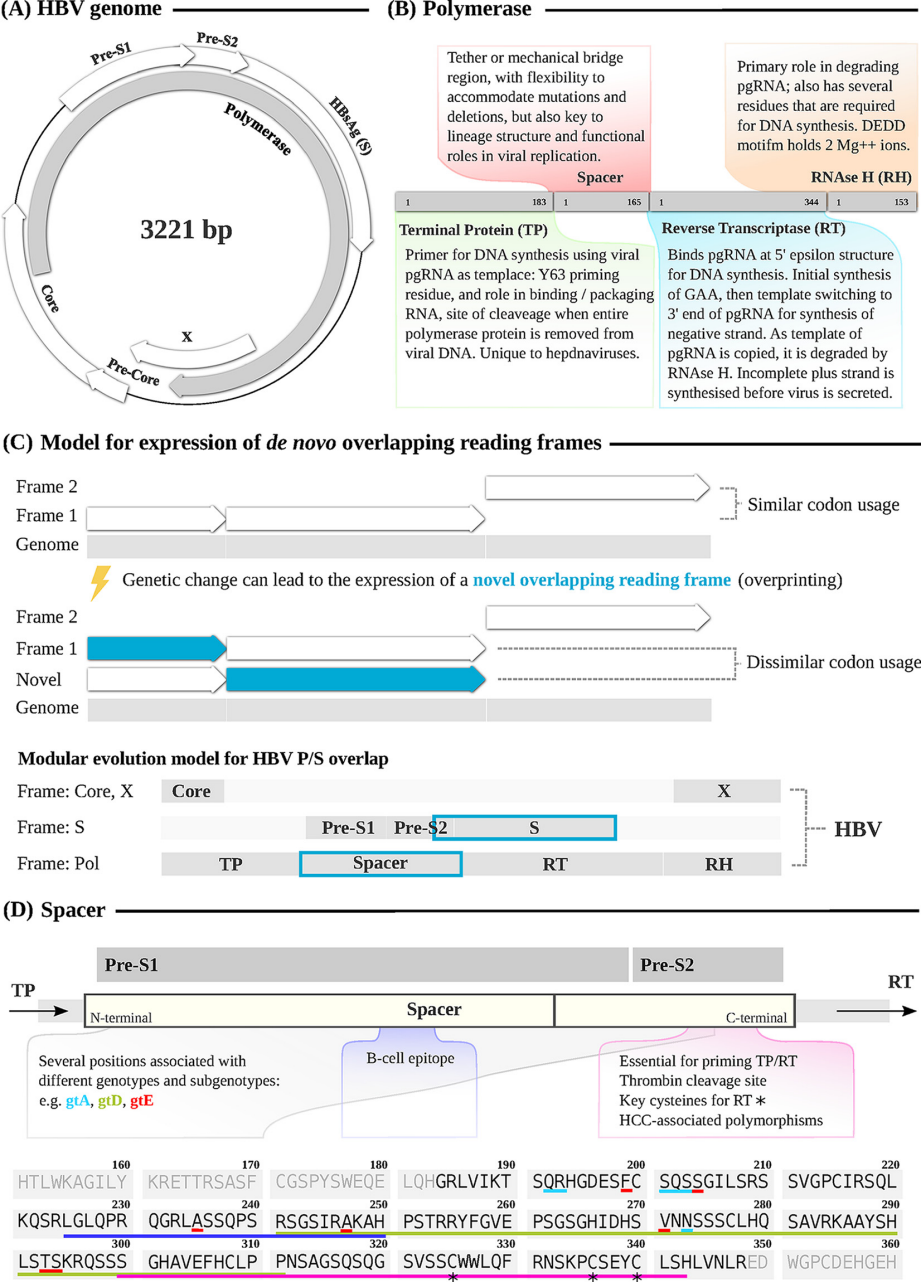
Genomic and functional roles of HBV spacer domain. (A) Structure of overlapping ORFs in the HBV 3.2-kb partially double-stranded circular genome. The entire length of surface (S) (400 aa) is encoded on an alternate frame within the length of Polymerase (P), making it the longest known overlap of any animal virus. (B) HBV P polyprotein, showing subdomains TP (terminal protein), spacer, RT (reverse transcriptase), and RNase H. (C) Model to illustrate overlapping reading frames (ORF) with different codon usage by overlapping genes and modular evolution theory due to imprinting. (D) Annotation of spacer domain (165 aa), illustrating examples of sequence polymorphisms associated with lineage (genotype), enzymatic function, and key functional outcomes. Deletions are best tolerated in the N-terminal 2/3 of the protein, while the C-terminal 1/3 contains the majority of residues with established functional roles. Numbering and sequence are based on genotype A reference genome accession no. X02763. Cysteine residues in the putative zinc finger DNA-binding motif are marked with a star at aa 325, 336, and 340 (equating to previously described positions 312, 323, and 327 in a genotype D strain). Coloured underlining of the sequence matches the annotation in the schematic lay-out above, as follows: turquoise, gtA; green, gtD; red, gtE; purple, B cell epitope; pink, key regions in C-terminus.

Hepatitis B virus (HBV) is a hepadnavirus responsible for 300 million chronic infections worldwide (1). A better understanding of HBV biology can provide improved insights into viral diversity and epidemiology and underpin refined approaches to treatment and prevention. The tiny genome (∼3.2 kb) is organized into four overlapping open reading frames (ORFs) (Fig. 1A), compressing a high density of genetic information (2–4). The HBV replication cycle is dependent on the viral polymerase protein (P), a polyfunctional protein composed of 4 subdomains: terminal protein (TP), spacer, reverse transcriptase (RT), and RNase H (Fig. 1B).

The spacer domain spans amino acids (aa) 184 to 348 of P (based on GenBank reference sequence X02763), sandwiched between TP and RT (5). Spacer has long been considered a dispensable subdomain, and the structure and function of this region therefore has not been investigated in detail. However, the question arises as to why a conserved spacer domain would have evolved and persisted if it had no specific function. More recent evidence is accumulating to suggest that spacer has a role in the HBV replication cycle, is subject to positive selection (as a result of evasion of therapy, vaccines, and host immune responses), is relevant for distinguishing between viral lineages, and can be associated with different outcomes of HBV infection.

Here, we synthesize evidence for the roles and significance of spacer and highlight gaps in our knowledge about this small but potentially influential region. We consider evidence from the older literature and assess how new insights can contribute to a refined understanding of spacer biology with practical relevance to pathophysiology, genotyping, determination of drug resistance, and sequence-based clinical stratification.

## DEFINITIONS AND TERMINOLOGY

The following terms are used here: adaptive evolution, changes in the frequency of beneficial variants and of deleterious variants due to selection; *dN/dS* ratio, the ratio of observed substitution rates of nonsynonymous versus synonymous genetic changes (*dN/dS* > 1 is generally interpreted as a proxy for the past occurrence of positive selection); negative selection, change in the frequency of variants within a population that may be harmful to fitness of the organism; neutral selection, stable frequency of variants within a population that have no effect on the fitness of the organism; nonsynonymous mutations, changes in the nucleotide sequence that result in changes in the amino acid sequence of a protein or introduce a premature stop codon; occult HBV, detectable HBV DNA in the absence of HBV surface antigen (HBsAg) in the serum; open reading frame (ORF), portions of DNA/RNA that contain no stop codons and can be translated to form amino acid sequences; phylogeny, the evolutionary history of a group of organisms or samples of the same organism; positive selection, changes in the frequency of variants within a population that may be beneficial to the fitness of the organism, e.g., replicative capacity, persistence, or transmissibility; synonymous mutations, changes in the nucleotide sequence that cause no change in the amino acids sequence of the protein due to codon redundancy.

## EVOLUTION OF HBV SPACER

Due to the intimate relationship between a pathogen’s genetic sequence and protein structure and function, here we consider the evolutionary pressures that may be relevant in driving sequence change in spacer and review the genotype specificity of observed polymorphisms.

### The spacer domain evolved through modular evolution.

Overlapping ORFs typically consist of an ancestral gene (encoding essential proteins) and a *de novo* gene that evolved subsequently through overprinting (6, 7), encoding accessory protein(s) (8, 9). A sequence analysis of 43 genera of RNA viruses infecting eukaryotes showed that most proteins created *de novo* are accessory proteins and are predicted to be fully disordered, similar to the spacer region of HBV P (8).

Methods developed for deltaretroviruses show that codon usage can distinguish ancestral genes (which have codon usage similar to nonoverlapping regions of the viral genome) from *de novo* genes (with codon usage very different from the rest of the genome) with high specificity (6, 10). Within HBV genomes, the entire length of surface (S) (400 aa) is on an alternate frame overlapping P (Fig. 1A) (11). However, analyses of P and S have been inconclusive in determining which of the two genes is ancestral, as both are essential for virus survival (11). Comparing the codon usage of the overlapping P and S ORFs has shown that codon usage of the entire overlapping frame (>1,000 nucleotides [nt]) compared to the nonoverlapping region is not significantly different for P and S (11). However, under a sliding window model, two regions of different codon usage can be identified, one in the 5′ third of the overlap and another in the 3′ two-thirds; these regions are conserved across hepadnavirus genomes (11). This suggests a modular evolution model for the P/S overlap, according to which the PreS1 domain, most of PreS2, and the RT domain of P are ancestral, while the spacer domain, the C-terminal third of PreS2, and the S domain evolved *de novo* by overprinting (Fig. 1C). This theory is consistent with an understanding of the functions of these domains, with essential roles of PreS1 in infectivity (12, 13) and RT in replication (14).

This proposed model of the primordial structure of the HBV genome may also explain why the P polyprotein contains RT and RNase H domains with retroviral homologues (15) but differs in having TP and a spacer domain. Congruent with the theory that spacer evolved later than other P domains, it is thought to have an intrinsically disordered structure (8, 16) (also see “Structure and Function,” below).

### Spacer is under positive selection.

It is generally understood that positive selection in one reading frame should be mirrored by negative selection in the overlapping frame (7, 17–20). Accordingly, calculation of the nonsynonymous to synonymous mutation ratio (*dN/dS*) of the S, core (C), and P genes of HBV can show that while S holds evidence of positive selection, C and P undergo negative selection (21). However, positive selection in S does not necessitate relaxed or negative selection in P; the frameshift between the two genes allows them to evolve independently, accommodating different evolutionary pressures (22).

Findings that *dN/dS* is >1 in significant segments of either P or S in the overlapping region and >1 in both genes in only a few key regions indicate that selection pressures fluctuate throughout the region (22). In the P/S overlap, the S ORF is shifted by 1 nt, meaning that the first codon position of P (p1) overlaps the third codon position of S (s3). Degeneracy within the nucleotide code means that changes to the first and second codon positions more frequently result in amino acid changes. Thus, adaptive evolution in P occurs through p1/s3 substitutions that cause an amino acid change in P but rarely in S, and adaptive evolution in S occurs through p3/s2 substitutions that cause an amino acid change in S but infrequently in P. Substitutions resulting in an amino acid change in both genes (p2/s1) are rare and contribute little to entropy across the sequence (22). Thus, while P and S genes are overall both under negative selection, to preserve their important roles in infectivity and replication, parts of both (particularly PreS and spacer) are subject to positive selection to evade the host immune system as well as to withstand anthropogenic pressures, such as vaccine-induced antibodies and antivirals (22) (discussed further in “Host-virus interaction and outcomes of infection,” below).

Although a high *dN/dS* ratio is observed in spacer compared to other domains in P and S, this does not conclusively determine whether spacer is undergoing true positive selection or is merely tolerant of nonsynonymous mutation (relaxed selection) (23). The variant degree (VD), a parameter used to evaluate the evolutionary selection of a sequence, is thought to negatively correlate with the importance of biological function. The VD of spacer and PreS2 of >20% contrasts with PreS1, S, and RT VD values of <10% (23), increasing the likelihood that spacer and PreS2 are undergoing relaxed selection (23). At the same time, PreS1, S, and RT are under stricter selection due to their critical roles in hepatocyte binding, cell entry (12, 13, 24–26), and genome replication and packaging, respectively (14, 27, 28).

### Spacer reflects HBV sequence diversity and lineage.

The spacer domain shows a high degree of nucleotide variation between different hepadnaviruses and between genotypes and subgenotypes of HBV (29–33), indicating that despite being evolutionarily newer than other regions of the genome, this domain has undergone extensive divergence over time (32, 33). This striking diversity may be enabled by the domain's disordered structure (16).

**FIG 2:**
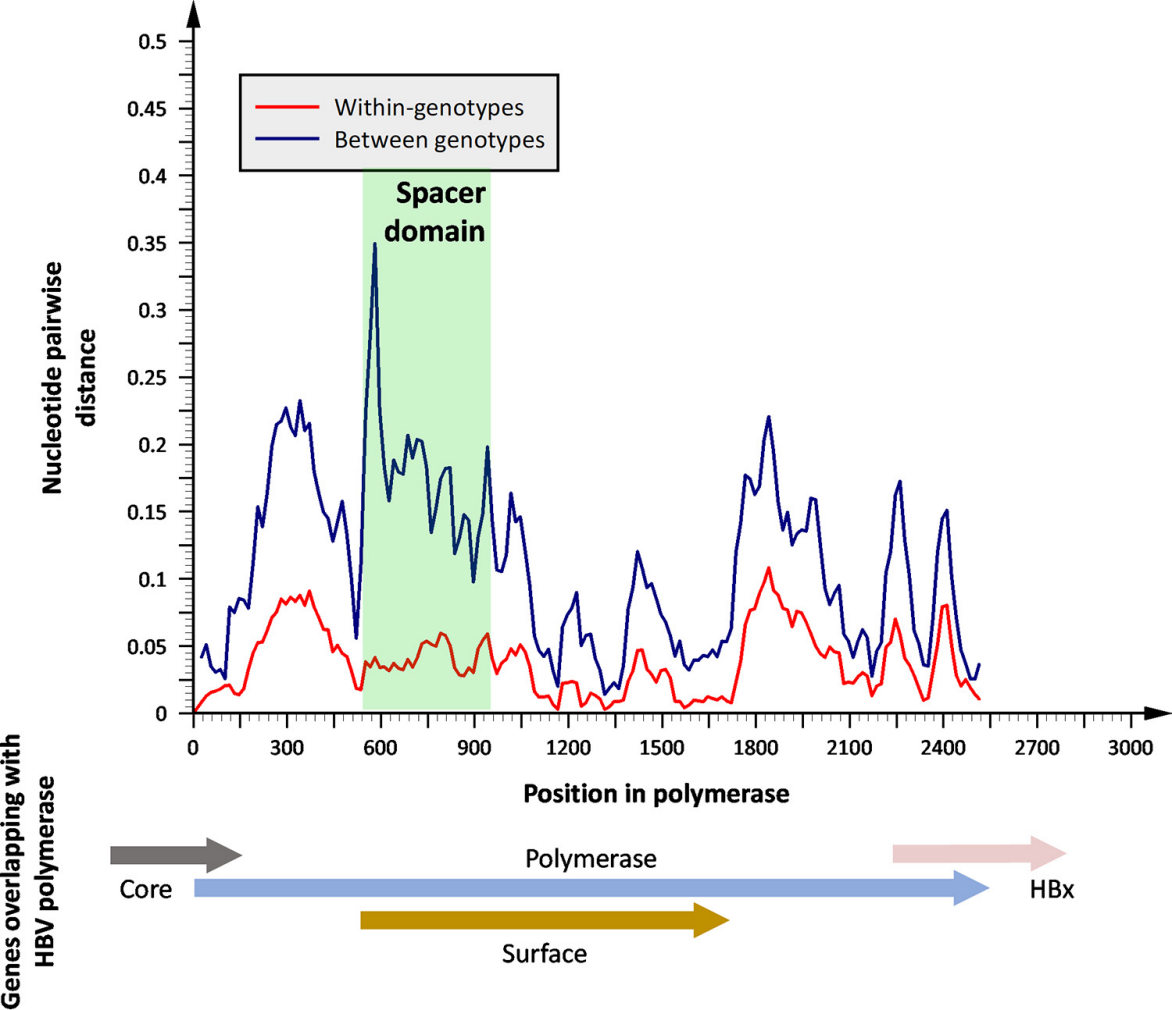
Nucleotide pairwise distances within and between genotypes in HBV polymerase. Representative HBV genotype sequences for genotypes A to I (129) were analyzed using a sliding window size of 100 bp and increments of 25 bp in SSE v1.3 (130). Putative genotype J was excluded, as regions of the genotype are thought to originate from nonhuman HBV sequences.

Pairwise analysis of nucleotide sequence space in P indicates that spacer holds low within-genotype diversity but high between-genotype diversity, contrasting with other regions of the protein (Fig. 2). If spacer is an inherently plastic region where mutations can accumulate without significantly affecting virus viability, one might expect variants to be stochastically distributed across phylogenies. However, there is accumulating evidence that spacer polymorphisms and deletions are sufficient to distinguish between HBV lineages (Table 1, Fig. 1D). Due to the frameshift between the P and S reading frames, the majority of nucleotide substitutions that result in amino acid changes in spacer will be synonymous in PreS1/2. Therefore, this phenomenon may not be fully explained through the well-recognized selection pressure acting on S (further discussed in “Genetic plasticity of HBV spacer,” below), and there may be incompletely understood selection pressures acting on spacer that result in lineage-specific motifs.

**TABLE 1:** Examples of evidence that spacer residues can be used to distinguish between HBV genotypes, subgenotypes, and geographic distribution of lineages^*a*^

HBV genotype	Location	Observation(s)	Reference
A, subgenotype A1	Brazil	Four amino acids in the polymerase, of which two (Pro18 and His90) are in spacer, and one in the core antigen were sufficient to determine sequence clustering with isolates from Asia but not with A1 isolates from Southern and East Africa	131
		These five amino acids supported Bayesian analysis, concluding that the Brazilian isolates formed part of the Asian-American clade of subgenotype A1 (posterior probability value, 0.996).	
A, subgenotype A1	Zimbabwe	Characteristic shared residues in PreS1, PreS2, and spacer associated with clustering of sequences as part of the South African A1 clade	132
		Spacer was relatively well conserved among the Zimbabwean isolates	
		Two spacer motifs (Ser9Gln10 and Glu18Ser19Phe20) could distinguish Zimbabwean isolates from clades found in other African and Asian countries where HBV/A1 is endemic but were shared with the South African HBV/A1 clade	
B and C	China	Genotype-specific mutations that distinguished genotypes B and C were found to be nonrandomly clustered in spacer	23
		56 of the 85 genotype-specific mutations were distributed in the 5′ half of the P/S overlap, of which 42 mutations led to amino acid changes in spacer, and 23 mutations caused amino acid changes in PreS	
		Only 29 mutations were present in the 3′ half of the overlapping reading frame, of which 13 caused residue changes in RT and 18 caused residue changes in S	
D, subgenotype D1/D4	Russia and the Baltic	Amino acids 58–128 of spacer contain subgenotype-specific mutations capable of reliably differentiating between D1 and D4 isolates	133
E	West Africa	Despite low intragenotype diversity of genotype E, the spacer region contained eight unique amino acid residues (Glu16, His21, Arg52, Asp55, Met64, Lys88, Asn110, and His111)	134
		A conserved genotype-specific signature motif was also discovered in PreS1	
Multiple	All	Deletions at the start of spacer (predicted to start at nt 2856 in the X02763 reference sequence) are highly conserved within genotypes	129
		Known conserved deletions in PreS1, Δ2861–3 in genotypes E and G, and Δ2854–2886 in genotypes D and J result in deletions at the start of spacer	

a Locations of the residues are given by numbering the start of the spacer domain as the first amino acid.

## GENETIC PLASTICITY OF HBV SPACER

### Spacer can accommodate insertions and deletions without functional impact.

P can accommodate large insertions and deletions in spacer and can maintain catalytic function, including protein priming, synthesis of the DNA minus strand, removing pregenomic RNA (pgRNA), and synthesizing plus-strand DNA (34, 35). In one of the first investigations of spacer function, 52 aa were deleted from duck HBV (DHBV) spacer with endogenous polymerase activity largely unaffected and without a quantifiable impact on DNA synthesis (5). Subsequent experiments have deleted large regions of spacer (e.g., amino acids 201 to 292) without affecting endogenous polymerase activity, with only residues 293 to 335 required to maintain enzymatic function (14). Furthermore, naturally occurring spacer variants with large in-frame deletions have replication competence similar to that of wild-type strains (36–38). For example, a sequence with a 69-amino-acid deletion in PreS1/spacer had wild-type (WT)-like polymerase activity, substantiating previous findings that the N-terminal portion of spacer is not essential for replication (36). Similarly, large insertions in spacer have been tolerated without an impact on DNA synthesis activity and RNA packaging capacity (33). Based on this evidence that spacer is capable of withstanding insertion, deletion, and substitution mutations without an impact on viral replication capacity, it is possible to conclude that spacer is a nonessential, or even entirely dispensable, subdomain of P that merely exists as a mechanical tether between TP and RT (5, 14, 23, 27, 33–35, 39–43). Table 2 provides a summary of spacer mutagenesis studies.

**TABLE 2:** Summary of HBV mutagenesis studies in spacer and the resultant phenotype^*a*^

Virus	Spacer residues	Phenotype	Reference
Deletions			
HBV	aa 175–300	No ability to engage in RNA binding, RNA packaging, or protein priming	69
HBV	aa 178–336	Reduced polymerase activity (70% of WT)	65
HBV	aa 179–257	No impact on replication capacity and RNA encapsidation	67
HBV	aa 196–291	Increased expression; decreased protein priming ability	62
HBV	aa 199–300	Incapable of RNA packaging; able to bind RNA and perform protein priming	69
HBV	aa 201–292	Decreased polymerase activity (40% of WT)	14
HBV	aa 201–292	Decreased polymerase activity (40% of WT); no impact on RNA packaging	34
HBV	aa 201–335	Impaired polymerase activity (<0.3% of WT)	14
HBV	aa 258–286	Capable of RNA encapsidation	67
HBV	aa 258–336	Incapable of replication and RNA encapsidation	67
HBV	aa 287–317	Incapable of RNA encapsidation	67
HBV	aa 293–335	Impaired polymerase activity (<0.3% of WT)	14
HBV	aa 293–335	Impaired polymerase activity (<0.5% of WT); decrease in RNA packaging efficiency	34
HBV	aa 300–334	Impaired priming function	39
HBV	aa 318–336	Incapable of RNA encapsidation	67
HBV	nt 2878–129	Increased stability of the P protein	66
DHBV	aa 307–356	No impact on polymerase activity	5
Substitutions			
HBV	C312A, C323A, C327A, C341A	Each mutation individually is incapable of RNA binding, RNA packaging, and protein priming	69
HBV	C312A, C323A, C327A	Each mutation individually is incapable of RNA encapsidation	67
HBV	C326R	No detectable replication activity	71
HBV	E245L	Reduced but still significant level of replication	71
HBV	R219S, R246G, G261R, N270S, A272S, R300H	Decreased replication efficiency	27
Insertions			
DHBV	Bacterial protein A inserted into spacer	No impact on DNA synthesis and RNA packaging activities	33
DHBV	12 nt at 1,212 bp (in frame)	No impact on replication capacity	40
DHBV	4 nt at 1,212 bp (frameshift)	Impaired replication capacity	40

a The majority of studies were in human HBV, with a small number also carried out using duck HBV (DHBV) as an animal model. Polymorphism locations are provided as cited in the primary studies, which may differ from reference genotype A sequence X02763. aa, amino acid residue; nt, nucleotide; bp, base pairs; WT, wild type.

### Spacer tolerates polymorphisms to accommodate positive selection in PreS.

Large parts of the S gene undergo positive selection to evade host immune responses, in contrast to the TP, RT, and RNase H domains, which are under negative selection to preserve important replicative functions (21). Most of the variability in S lies in the PreS domains, the “a” (major antigenic) determinant, and the C terminus (44). Positive selection in S occurs almost exclusively in known T and B cell epitopes, suggesting adaptive evolution to evade natural or vaccine-mediated immune responses (44) (also see “Host-virus interaction and outcomes of infection,” below). Due to the frameshift between the overlapping P and S reading frames, many nonsynonymous mutations in PreS (p3s2) result in synonymous mutations in spacer. Thus, spacer may mediate the conflict between diversifying and constraining forces in S and P, respectively. However, most positive selection sites in P are concentrated in spacer, indicating that spacer is not undergoing merely neutral selection or random evolution but may in fact be important for the HBV replication cycle beyond a role facilitating changes in PreS (44).

## STRUCTURE AND FUNCTION

### Spacer is characterized by a disordered protein structure incorporating protease digestion sites.

Spacer's secondary and tertiary protein structure has not been elucidated, and there is a growing recognition that spacer is likely to be an intrinsically disordered protein region (IDPR) (16), together with overlapping pre-S regions with which it overlaps (45). IDPRs can deliver important biological roles even in the absence of a stable physical structure; indeed, conformational flexibility may itself be a functional attribute, allowing them to perform “impossible tricks” that would not be possible for a molecule with a stable, rigid structure (46). Such regions may have specific characteristics (for example, in diversity, charge, and polarity) and are common in viruses, with potentially key roles in protein-protein interaction (PPI) networks (47, 48).

Crystal structures have not been solved for HBV P, with challenges including the multidomain nature of the polyprotein, the possibility of more than one structural configuration, and its hybrid nature incorporating regions that are structurally ordered (such as RT), combined with IDPRs (46, 49). In addition to spatial flexibility, spacer’s disordered structure may render it highly sensitive to protease digestion (42, 50). Spacer has a cleavage site for thrombin, deduced by adding thrombin to Pol proteins of various lengths, and can be cleaved by cellular proteases in rat hepatocytes and human hepatoma cells *in vitro* (51, 52). Critically, these cleavage sites are conserved across hepadnaviridae (52), providing some evidence for a functional role. In theory, protease digestion could separate functional TP and RT/RNase, facilitating movement of the polymerase down the RNA while TP remains bound at the 5′ end (5).

However, while multiple studies have observed smaller molecular forms of P *in vitro* using a variety of assays (5, 52–54), and proteolytic cleavage is a known mechanism of protein activation in other viral proteins (55–57) (including the generation of HBe antigen [HBeAg] through cleavage of the precore/core fusion protein [58]), there is no protease requirement for functional activity of P (59, 60). The intact polyprotein demonstrates RT activity *in vitro (*59), and full-length P can be found intact in virions while carrying out the essential function of genome encapsidation (60). Furthermore, there is no evidence to suggest that protease digestion plays a role in later stages of the HBV replication cycle, such as cccDNA formation (52). A continued linkage model is based on evidence that TP and RT remain connected during replication, explaining second-strand initiation and conversion from a linear to a circular genome (5, 42, 43).

### Spacer provides protein flexibility.

Spacer may be crucial in providing flexibility for the TP and RT domains to assume the structural conformations needed to perform diverse functions, including RNA 5′ epsilon binding, pgRNA encapsidation, and DNA synthesis. For these events to occur, P must switch from a stable (inactive) configuration to its active state, the kinetics of which may be enabled by spacer and facilitated by a variety of host cellular chaperones (44), such as heat shock protein 40 (hsp40), hsp70, and hsp90 (61–64), although a direct host-virus interaction has not been elucidated. This flexible, dynamic structure is typical of an IDPR, with interplay between subdomains facilitated by interaction with other viral and host proteins and by characteristics of the environment, such as pH and temperature (46).

### Role of spacer in P function.

Mutagenesis studies (Table 2) have investigated the role of the spacer domain in the function of P. Performing experiments *in vitro* has enabled researchers to isolate the effect of mutations on P function, preventing any confounding effect that the mutations in the overlapping reading frame have on protein S. Deletion of the spacer domain has generated conflicting evidence, demonstrating both reduced (65) and increased (66) DNA-dependent DNA-polymerase activity. However, increased activity following spacer deletion may relate to higher expression and stability of the P protein without spacer (62, 66). In contrast, replacing the spacer domain of a less efficiently replicating isolate with the domain of a more efficiently replicating isolate has led to substantial increase in replication efficiency (27); the authors explained this increase with a point mutation at residue 300, part of the minimal portion of the spacer domain, although the isolated role of residue 300 remains unclear. Indeed, experiments with spacer deletion mutants have suggested differing significance of the N-terminal and C-terminal regions (Fig. 1D). Deletion of the N-terminal two-thirds of spacer does not affect RNA encapsidation, replicative capacity, or pgRNA availability (34, 67). Conversely, deletion of the C-terminal third of spacer results in a drastic decrease in RNA packaging efficiency (34, 67), with residues 300 to 334 being essential for the priming function of both TP and RT domains (39), suggesting it is required for replication competence (14).

In addition to the C-terminal region, there is also evidence that other upstream residues of spacer play a functional role. In a deletion mutant study, the TP domain did not require spacer to function, but maximal activity of TP required the N-terminal part of spacer (39). Another study deleted the central part of spacer (aa 196 to 291) and demonstrated that although the truncated P was more readily expressed than full-length P, it could not engage in protein priming (62). A successful interaction between truncated P and the RNA epsilon element could, however, be reconstituted, suggesting that while the spacer deletion mutant remains capable of binding RNA, mere physical binding is not sufficient for a functional protein–RNA interaction (62). An intact spacer domain therefore may be needed for conformational changes required for protein priming or interaction with essential cellular chaperones.

Three cysteine residues in the C-terminal region of spacer (positions 312, 323, and 327, based on a genotype D alignment) (Fig. 1D) have been established as essential for RT activity (29, 67–69). Together with a fourth cysteine in the N terminus of RT, these residues form part of a putative zinc finger DNA-binding motif (69, 70) and are conserved across mammalian hepadnaviridae (67). A C327A substitution in the C-terminal portion of spacer is lethal for HBV, abolishing endogenous polymerase activity, suggesting that spacer has a direct role in replication or an essential role in the folding of P to enable replication (71). Nearly 2 decades after this original research, the essential role of these cysteine residues in pgRNA encapsidation and for binding of P to the 5′ epsilon stem-loop of pgRNA was confirmed using alanine-scanning mutagenesis (67). Cotransfection of mutant and WT P in a 3:1 ratio did not have a dominant-negative effect: the mutant protein does not bind pgRNA or incorporate into nucleocapsids, and WT P binds and carries out replication (67). Mutating the three C-terminal cysteine residues produces variants that are completely defective in 5′ epsilon binding, protein priming, and RNA packaging. Therefore, overall this putative zinc finger domain is essential for RT catalytic activity (29, 68).

It is noteworthy that data from *in vitro* HBV replication models have been limited due to reliance on heavily adapted cell culture systems and with spacer modifications (e.g., green fluorescent protein insertions and various deletions) that may interfere with enzymatic function, making it difficult to cross-compare data from different studies. More recently developed culture approaches offer the potential for studying HBV replication more reliably (72, 73). Furthermore, while findings from animal models should always be extrapolated with caution, DHBV and similar animal models are considered reliable for use in studying viral entry, polymerase function, and immune response, although less reliable to study the clinical manifestations of HBV, such as cirrhosis and hepatocellular carcinoma (HCC) (74).

## HOST-VIRUS INTERACTION AND OUTCOMES OF INFECTION

Spacer has been determined to contain the highest number of positively selected sites of any domain in the HBV genome. Maximum likelihood estimates of codon selection suggest spacer is at the center of a complex epistatic network coordinating clusters of mutations involved in virulence, immune escape, and drug resistance (16).

### Spacer mutations associated with HCC.

The incidence of HCC, disease progression, and treatment outcomes are influenced by a complex interplay of host, viral, and environmental factors. Certain viral genotypes (including C and F) are associated with a higher lifetime risk of HCC (75–80), and mutations in spacer, as well as other regions, have been associated with HCC (75, 76, 81–83). Next-generation sequencing has identified multiple HCC-associated single-nucleotide variants (SNVs), concentrated in PreS1 and spacer (84). A mutation at nt 31 was significantly associated with higher alpha-fetoprotein levels, larger tumor size, and shorter postoperative survival (85). However, the role of these SNVs in hepatocarcinogenesis remains poorly understood, as it is unclear if oncogenic activity is driven by changes to the PreS1 and/or spacer function (84, 86).

PreS deletions, which also result in deletions within the overlapping spacer, have been reported as independent risk factors for HCC (81–83, 87–89), potentially as a result of accumulation of the misfolded mutant surface antigen causing endoplasmic reticulum stress (87, 90–93). A double-spliced (2.2-kb) HBV variant isolated from liver tissue had deletions spanning most of spacer and PreS2, and parts of PreS1, S, RT, and TP (94), and the 2.2-kb variant is increased in HCC tissue compared to peritumor tissue (95). Although the double-spliced variant is not replication competent, adding it to the full-length version resulted in dose-dependent enhancement of replication efficiency (94). Splice variants therefore might contribute to the increased and persistent HBV replication that leads to HCC in some patients (96, 97). As the S and P proteins are translated from different RNA transcripts, functional studies of these deletions have only considered the impact of the deletions in S, but it is possible the corresponding deletions in spacer also contribute to disease progression.

### Spacer mutations associated with OBI.

Mutations in spacer have also been described in the setting of occult HBV infection (OBI), defined as detectable HBV DNA in the absence of HBsAg in the serum (the marker of active ongoing infection) (98). OBI can reactivate to cause hepatitis flares (99, 100), can be associated with HCC development (101–103), and can be a reservoir for transmission (104, 105). In a study reporting 235 OBI-associated mutations, 151 were in P and 27 in spacer, reflecting immune evasion and leading to decreased viral replication and reduced immune activation (106). However, the biological and clinical significance of specific polymorphisms remains to be clearly elucidated.

### Escape from the adaptive immune response and vaccine-mediated immunity.

P is an important target of the cellular and humoral immune response (107–115), harboring epitopes for antibodies and CD4^+^ and CD8^+^ lymphocytes (30, 116–118). While some regions of P are functionally conserved, variation elsewhere in the polyprotein can contribute to immune evasion. Positive immune selection pressure acts on parts of P, particularly spacer, both in concert with PreS and independent of the overlapping S gene (22, 44). In a study of HBV genotype D, 13 out of 15 sites under positive selection were located in spacer, and some overlap was detected between positively selected sites in spacer and in PreS, indicating that positive selection can colocate in overlapping genes (44).

One B cell epitope in spacer (aa 225 to 250) may explain some of the sequence diversity of the domain (30) (Fig. 1D). Sequence analysis of P and S genes in vaccine escape mutants among HBV-vaccinated children (119) detected amino acid substitutions that affect B and T cell epitopes, including 8 amino acid substitutions in this B cell epitope in spacer (119). However, disaggregating the specific influence of individual mutations in mediating vaccine escape requires further efforts.

### Spacer mutations in the setting of drug resistance and to preserve viral fitness.

There is some evidence that spacer accommodates compensatory mutations to ameliorate a fitness detriment caused by mutation(s) elsewhere in the viral genome. Due to spacer's proximity to key functional residues in TP and RT, it is possible that amino acid mutations in spacer affect the catalytic activity of P and therefore could fine-tune or restore changes imposed by other mutations (e.g., see reference 44). A study of covariance in genotypes B, C, and D found that polymorphisms were concentrated in the spacer and PreS1, suggesting a coevolutionary relationship between these sites and sites elsewhere in the genome (120).

Lamivudine (3TC) resistance mutations in the YMDD motif of RT, which negatively affect replicative ability, can be restored by compensatory mutations in the putative zinc finger subdomains (121), including spacer’s C-terminal cysteines (29, 67–69). Other mutations can also be relevant in driving resistance; for example, RT (A181T) and spacer (S331C) polymorphisms together result in a decrease in 3TC susceptibility (122). A181T, S331C, and A181T+S331C mutants were 82%, 94%, and 96% replication efficient, respectively, compared to the wild type (122). Although the effect is modest, it is possible that the spacer mutation coevolved to restore a minor fitness detriment caused by the RT mutation (122), since residue 331 is within the portion of the spacer important for RT function (39). Furthermore, after 3TC discontinuation (when the mutant strain became undetectable), 3TC readministration has been associated with the reappearance of both mutations (122). A spacer deletion arising in an adefovir-treated patient is of uncertain significance in drug resistance (36), while selection of a mutation (A300E) has also been reported from a patient with clinical evidence of entecavir resistance, although this polymorphism did not alter *in vitro* resistance or replication capacity (123). The sparse evidence base for treatment-associated mutations in spacer is related to the lack of routine investigation of drug resistance in clinical practice and focus only on RT if sequencing is undertaken (29).

A study of duck HBV provided evidence of spacer's potential reactivity to mutations elsewhere in the genome, tolerating changes in its own sequence to overcome a fitness detriment by introducing insertions into the distal PreS (40). *In vivo*, in-frame mutants retained the inserted nucleotides, while frameshift mutants either reverted to wild type or selected a deletion in spacer/PreS that shifted the frame back to normal, restoring infectivity and secondary protein structure and demonstrating compensation for a fitness cost by deleting a specific nonessential portion of the genome (40).

## FUTURE DIRECTIONS AND TRANSLATIONAL APPLICATIONS

HBV sequencing has not been widely applied due to lack of a mandate for genotyping in clinical guidelines (e.g., see reference 124) and the typically low viral loads of many chronic infections (125). However, as sequencing methods are improved and there is increasing interest in the application of sequence data, opportunities will arise to enhance insights into viral diversity and its impact on disease outcomes (4, 126). Sequencing repositories contain an untapped resource for spacer data by way of sequences that have been generated for the S gene but could be mined for analysis of spacer (Fig. 1A).

To date, structural insights for P have been extrapolated from the homologous regions of the HIV polymerase (127). Determination of crystal structures may provide insights into the interactions between protein subdomains, including better understanding the role of spacer in maintaining integrity, supporting the viral replication cycle, and accounting for diverse outcomes of infection. The oncogenic mechanism of viral variants associated with HCC remains to be elucidated, but pathogen genomics may be relevant for future personalized medicine approaches for cancer surveillance or risk assessment. More research is needed to determine the specific protein-protein interactions between spacer and host chaperones and restriction factors. Ultimately, driving this agenda is important to inform clinical practice, for example, through using viral sequence to stratify patients for surveillance and/or treatment, to guide public health interventions, and to inform the development of new therapeutics (128).

## CONCLUSIONS

Spacer is a small but highly diverse and versatile domain, with features of an IDPR. Earlier studies assumed spacer was a dispensable region, and it is doubtless true that there is some redundancy, particularly in the N-terminal portion. However, evidence has also accumulated for important evolutionary and biological roles, more focused in the C-terminal third, which play essential roles in RNA binding and packaging, protein priming, and reverse transcription, with a dynamic structure that supports protein function, including potentially coordinating host-virus interactions. Further research is required to more definitively elucidate the functions of specific spacer residues.

Spacer sequence variation between and within mammalian hepadnaviridae, as well as between genotypes and subgenotypes of HBV, is highly lineage specific, overturning the hypothesis that spacer is diverse because of low functional or evolutionary importance. Rather, spacer may play a crucial role in the evolution of both the P and the overlapping S genes, influenced by pressure from exposure to drugs, vaccines, and the host immune response. Through the increasing use of whole-genome sequencing data and trends in personalized medicine, we are in an era of opportunities to further unravel the role of spacer in HBV evolution, epidemiology, and pathogenesis, with potentially important translational implications.
